# Subwavelength Grating Double Slot Waveguide Racetrack Ring Resonator for Refractive Index Sensing Application

**DOI:** 10.3390/s20123416

**Published:** 2020-06-17

**Authors:** Nikolay Lvovich Kazanskiy, Svetlana Nikolaevna Khonina, Muhammad Ali Butt

**Affiliations:** 1 Department of Technical Cybernetics, Samara National Research University, 443086 Samara, Russia; kazansky@smr.ru (N.L.K.); khonina@smr.ru (S.N.K.); 2 Institute of RAS-Branch of the FSRC “Crystallography and Photonics” RAS, 443086 Samara, Russia

**Keywords:** single-slot waveguide, double slot waveguide, subwavelength grating single slot waveguide, subwavelength grating double slot waveguide, racetrack ring resonator, refractive index sensor

## Abstract

In this paper, a racetrack ring resonator design based on a subwavelength grating double slot waveguide is presented. The proposed waveguide scheme is capable of confining the transverse electric field in the slots and the gaps between the grating segments. This configuration facilitates a large light–matter interaction which elevates the sensitivity of the device approximately 2.5 times higher than the one that can be obtained via a standard slot waveguide resonator. The best sensitivity of the design is obtained at 1000 nm/RIU by utilizing a subwavelength grating double slot waveguide of period 300 nm. The numerical study is conducted via 2D and 3D finite element methods. We believe that the proposed sensor design can play an important role in the realization of highly sensitive lab-on-chip sensors.

## 1. Introduction

Silicon wire waveguides (WGs) are proficient in strong optical confinement into the narrow-area (<500 × 500 nm) due to the high index contrast in the Silicon (Si)/Silica (SiO_2_) system. This allows sharp bends with a radius as small as 5 µm resulting in miniaturized photonic integrated circuits. Recently stated, a slot WG is a light guiding structure that can intensify the optical field in a nanoscale void (slot) of low refractive index material (can be air, n = 1.0) inserted between higher refractive index material (rails) [1,2]. Considering a high index–contrast interface, Maxwell’s equations suggest that the corresponding electric field must experience a large discontinuity with much higher amplitude in the low index region to comprehend the continuity of the normal part of electric flux density. A Si/SiO_2_ platform is chemically secure and appropriate for on-chip gas [3,4] and bio-sensing [5] applications.

Subwavelength gratings (SWGs) can be used to craft an artificial media built on a microscopic scale to obtain the desired macroscopic behavior. A subwavelength grating for the silicon-on-insulator (SOI) platform can be created by the periodic arrangement of high index material (Si) and low index material (SiO_2_) or other low index materials. Such periodic structures surmount diffraction and functions as a uniform medium provided that the periodicity does not follow Bragg’s coupling condition criterion to other confined or radiative modes [6]. SWGs are commonly used as antireflective coatings [7], planar mirrors [8], broadband mirrors [9], fiber-chip couplers [10,11], modulators [12] and sensors [13,14], among others. Light excites a Bloch mode in SWG WG, with a core comprised of a periodic arrangement of Si and SiO_2_ segments. In theory, this mode can propagate through the periodic WG segment with no losses incurred by diffraction into radiative or cladding modes. By manipulating the pitch, width and duty cycle of the SWG WG, the effective index of the medium can be locally engineered.

Several sensor designs based on SWG WGs are proposed in previous studies, which include ring resonators [15], grating [16] or photonic crystal [17]. Besides, there are other highly sensitive plasmonic sensor designs based on metal-insulator-metal WGs also presented [18,19,20,21]. The sensitivity of such devices is determined by the interaction between the electric field and the ambient medium, which can be improved by increasing the light–matter interaction. For conventional WGs, such as ridge or rib [22,23], due to the high index contrast of Si (n = 3.48 @1550 nm) and SiO_2_ (n = 1.44 @1550 nm), the utmost mode field power is confined in the WG core. One way to increase the sensitivity for TE polarized light is to decrease the core thickness of the WG which enhances the evanescent field that contributes to better light–matter interaction. A bulk sensitivity of 100 nm/RIU is achieved [24], as demonstrated by TalebiFard et al., with 90 nm thick SOI strip WG.

In this paper, we analyzed four different configurations of slot WG, such as single slot waveguide (SSWG), double slot waveguide (DSWG), subwavelength grating single slot waveguide (GSSWG) and subwavelength grating double slot waveguide (GDSWG) using the finite element method (FEM). The modal characteristics of the WGs are studied in the first part of the paper and the dominance of SWG WGs on the standard slot WG schemes is manifested. In the second part of the paper, racetrack ring resonators based on the above mentioned WG schemes are studied, revealing an extraordinary sensing capability of the sensor utilizing SWG WGs, which outclasses the previously reported works.

## 2. SWG Slot WG Geometry and Theory

Bloch–Floquet formalism shows how electromagnetic waves are propagated in periodic media [6]. Based on the wavelength, propagation can be categorized into three wavelength regions for a specified grating period (ᴧ): (i) The sub-wavelength region in which the wavelength to period ratio is λ/ᴧ>2·n_eff_. This correlates to the wavelength range greater than the Bragg wavelength and the WG behaves as a standard WG. The periodic structure, in this case, retains a true lossless mode [25]. (ii) The wavelength spectrum is analogous to the photonic bandgap in which Bragg reflections take place. (iii) The wavelength range shorter than the Bragg wavelength, where the Bloch wave is leaky and part of the energy, is radiated out of the WG and the propagation loss is determined by reflection and diffraction at the segment boundaries caused by the high refractive index difference between air and silicon.

By having ᴧ<<λ, the mode is lossless since the reflection and diffraction effects are concealed. This is analogous to the distribution of electrons in periodic potentials, as in semiconductor materials. SWG WGs are attractive because they allow tailored propagation properties by varying the period (*l_grat_+d*), WG width (*W_rail_*) and WG height (*H_rail_*). Our suggested SWG WG scheme is based on slot WG, which is divided into periodic slot segments with linewidth (*l_grat_*). A slot WG has recently been implemented as a novel WG structure to confine and direct light in a nanometer-sized low refractive index material. A slot WG consists of two strips (rails) of a high-index material separated by a narrow low-index (slot) region. Thanks to its outstanding features, the slot WG is highly attractive for sensing applications [26].

In the case of a typical slot WG, the light is confined at the interface between highindex–contrast materials in the *xy*-plane by the electric field discontinuity, and high optical intensity can be obtained in the slot. The periodicity in the z-direction (*n*^2^(*z*) *= n*^2^(*z + l_grat_ + d*)) guarantees that the wave vector *k_z_* is still preserved. An electromagnetic solution takes the following form, according to the Bloch (or Floquet) theorem:

(1) $$E=E_{K}\left(x,y,z\right)e^{-iKz}$$

where *K* is the Bloch wavenumber and *E_K_*(*x,y,z*) is a periodic function with period (*l_grat_+d*), so that *E_K_*(*x,y,z*) *= E_K_*(*x,y,z + l_grat_+d*).Similar to the dispersion relationship for typical WGs, the dispersion relation for SWG WGs is *ω = ω*(*K*). Depending on the spectral regime, the Bloch wave vector *K* can either be real or complex. If *K* is real, the Bloch wave intensities will be a periodic function of position in the medium and propagate with no loss. There are analytical solutions for a layered structure with uniform material properties in the *xy*-plane, but not for the case of index driven modes (vertical and lateral confinement) and computational techniques should be used.

**Figure 1:**
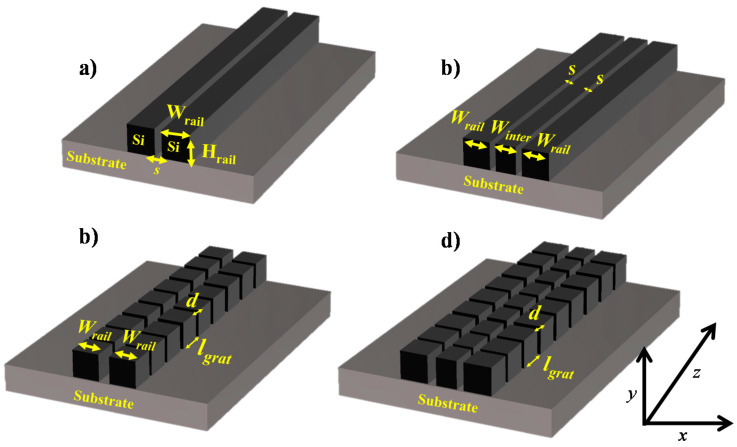
Schematic of (**a**) Single slot waveguide, (**b**) Double slot waveguide (**c**) Subwavelength grating single slot waveguide, (**d**) Subwavelength grating double slot waveguide.

Figure 1 represents the schematic of an SSWG, DSWG, GSSWG and GDSWG. The structure is designed on a silicon-on-insulator (SOI) platform with a 220 nm thick top silicon layer (h) and a 3 um thick buried oxide (BOX) layer. The geometric parameters of the WGs are tabulated in Table 1.

The WG models are simulated using the 3D finite element method (FEM) based model in COMSOL Multiphysics 5.1. The E-M wave frequency domain (emw) was used as a physics interface. In COMSOL simulations, the sub-domains in the WG cross-section are divided into triangular mesh elements with a “very fine” mesh grid size for the entire WG design. The meshing relies on the precision of the solution and the computational capacity of the system used. The meshing used in this work provides precise simulation results based on our system processing speed. For wave propagation systems, a domain with open computational domain boundaries is necessary as it allows the EM wave to travel without any reflection. The open geometry is determined by assigning scattering boundary conditions (SBC) at the outer edges of the simulation window.

**Figure 2:**
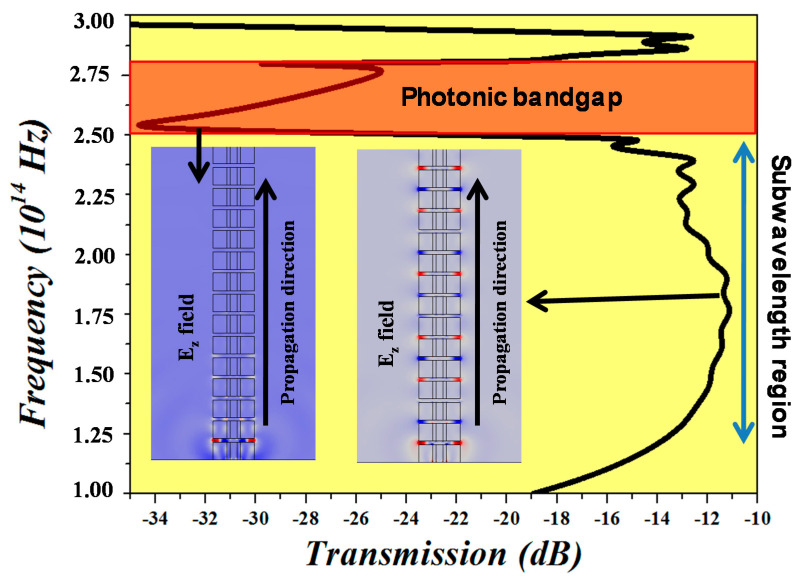
The transmission spectrum of GDSWG which is divided into two regions—photonic bandgap and subwavelength region. Inset of the figure shows the E_z_ plot of a WG in both the regions.

The transmission spectrum of GDSWG with ᴧ = 300 nm (Duty cycle (η) = 0.833) is plotted over a wavelength range of 1000–3000 nm, as shown in Figure 2. The transmission spectrum is determined using the following expression: $Transmission\;\left(dB\right)=10\times log\frac{P_{out}}{P_{in}}$, where *P_out_* and *P_in_* are the output power and input power, respectively. The Bloch mode propagates in the direction normal to the periodic structures with a wavevector k = 2π/λ and a temporal frequency ω. The resonant region where ᴧ~λ/2 and where the wave is not transmitted is referred to as a photonic bandgap. However, in the subwavelength regime *ᴧ < λ*/2, the structure behaves as a homogeneous effective medium with an effective index *n_eff_ = c(k/ω),* where c is the velocity of light. The mode is lossless since it conceals the reflection and diffraction effects. The wavelength region between ~1070–1220 nm is photonic bandgap for the designed WG, whereas the region over ~1240 nm is the subwavelength region which will be used as an operational wavelength for the ring resonators presented in Section 4.

## 3. Mode Sensitivity Analysis of Single and Double Slot WG

**Figure 3:**
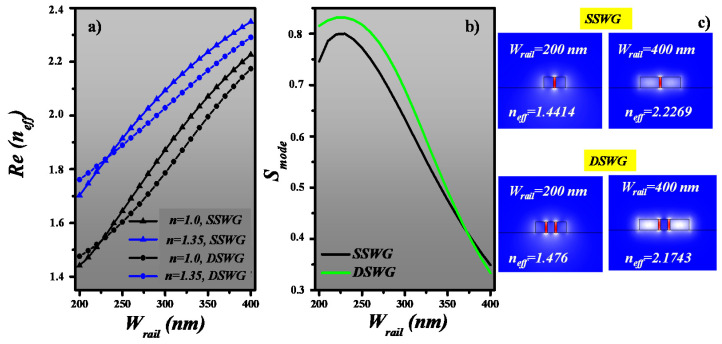
(**a**) Real part of the effective refractive index of SSWG and DSWG, (**b**) Mode sensitivity analysis, (**c**) E-field distribution in SSWG and DSWG at W_rail_ = 200 and 400 nm.

In this section, the mode sensitivity (*S_mode_*) analysis of SSWG and DSWG is performed, which reveals the dominance of double slot WG in terms of light–matter interaction. The effective refractive index *(n_eff_*) of SSWG and DSWG is calculated at λ = 1550 nm by varying the W_rail_ in the range of 200 to 400 nm. The remaining parameters, such as s and W_inter_ (for DSWG), are fixed at 50 and 150 nm, respectively. The real part of *n_eff_* of SSWG and DSWG at n = 1.0 and 1.35 is plotted in Figure 3a, which increases as W_rail_ increases and allows the formation of dielectric mode in the rail. *S_mode_* is calculated using the following formula:

(2) $$S_{mode}=\frac{\Delta n_{eff}}{\Delta n}$$

where *Δn_eff_* is the change in effective refractive index due to change in the refractive index of the ambient medium (Δn). From Figure 3b, it can be seen that *S_mode_* of both SSWG and DSWG is strictly dependent on W_rail_, which decreases as W_rail_ increases. At W_rail_ = 230 nm, the maximum *S_mode_* of 0.8 and 0.831 for SSWG and DSWG is obtained, which is due to the maximum overlap of the evanescent field tail in the slot region, respectively. However, at W_rail_ = 400 nm, the high index cladding region is large enough to support dielectric mode. As a result, the mode confined in the low index slot region splits up and the mode power in the slot is reduced, which brings to the *S_mode_* of both WGs to ~0.34. The E-field distribution of the mode in SSWG and DSWG at W_rail_ = 200 and 400 nm is shown in Figure 3c.

**Figure 4:**
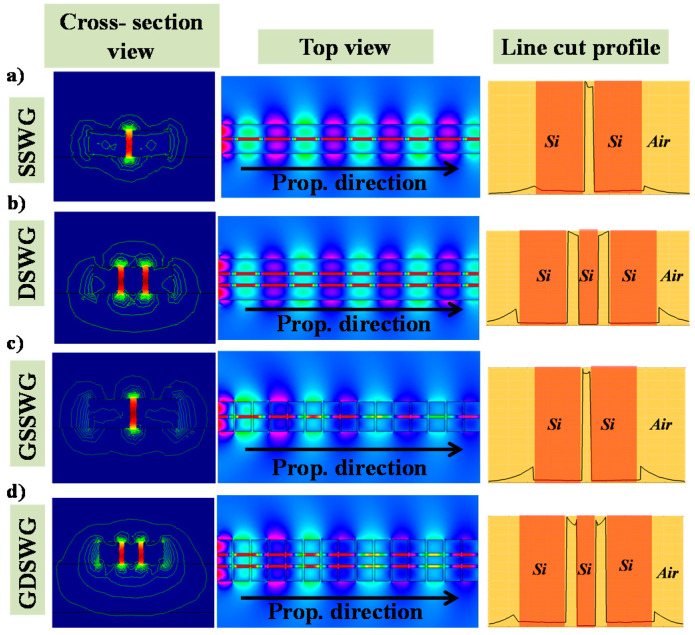
E-field distribution in the cross-sectional view, top view and line cut profile of electric field intensity of (**a**) SSWG, (**b**) DSWG, (**c**) GSSWG, (**d**)GDSWG.

From *S_mode_* analysis, it is evident that double slot WG configuration is more sensitive than single slot WG. Along with the low index slot region, SWG WGs has air gaps between silicon segments along the propagation direction of light. The propagating mode power is confined in the slot as well as in the gaps between the grating segments. This combined power exposed to the ambient medium can result in an elevated *S_mode_*, which is proven in Section 4. The E-field distribution of the TE-polarized light in SSWG, DSWG, GSSWG and GDSWG is calculated at λ = 1550 nm, which lies in the subwavelength region. The geometric parameters such as H_rail_, W_rail_, s, W_inter_ are fixed at 220, 200, 50 and 150 nm, respectively. The period of SWG WGs is chosen to be 300 nm with η = 0.833. The cross-sectional view (first column), top view (second column) and line graph (third column) of the WGs with E-field distribution are displayed in Figure 4. The cross-section view is taken in the middle of the WG (for SWG WGs, it is taken in the middle of the silicon grating segment i.e., *l_grat_*/2), whereas the top view is taken at *H_rail_*/2. The linecut profile of electric field intensity (|E|^2^) is taken in the middle of the respective WGs. There is no evident difference in the E-field distribution of the propagating mode in the standard slot WGs (Figure 4a,b) and SWG slot WGs (Figure 4c,d).

The WG structures are optimized at λ = 1550 nm to obtain a single-mode operation with high sensitivity to the waveguide cladding, while meeting the minimum feature size limitations imposed by the fabrication constraints (~40 nm gap size using electron-beam lithography and reactive-ion etching). The mode sensitivity can be directly derived from the dispersion diagrams corresponding to different cladding refractive index values. It is worth noting that the sensitivity of mode can be related to the overlap integral in the cladding (*Γ_c_*) [15], which is the sum of the overlap factors of the following three regions:

1. For SSWG and DSWG, the slot region/s between silicon grating segment with a volume of $s\times WG_{length}\times H_{rail}$ and $2\times s\times WG_{length}\times H_{rail}$, respectively, whereas for GSSWG and GDSWG, the slot region/s between two silicon segments (*Γ_slot_ and Γ_slots_*) with a volume of $s\times l_{grat}\times H_{rail}$ and 2 × ($s\times l_{grat}\times H_{rail})$, respectively.
2. For GSSWG, the gap region between the periodic silicon segments (*Γ_gap_*) with a volume of $\left(2\times W_{\mathrm{rail}}+s\right)\times d\times H_{rail}$. In the case of GDSWG, it is calculated as $\left(2\times W_{\mathrm{rail}}+W_{\mathrm{inter}}+2\times s\right)\times b\times H_{rail}$.
3. The remaining upper cladding medium (*Γ_uc_*).

For the SWG WGs, the electric field intensity varies periodically along the propagation direction. Consequently, *Γ_subregion_* can be calculated by integrating the intensity over the volume of subregion (*slot, gap, upper cladding*) in a single unit cell volume with a period (ᴧ) by using the following formula:

(3) $$\Gamma_{\mathrm{subregion}}=\frac{{∭}_{\mathrm{subregion}\;}{\left|E\left(x,y,z\right)\right|}^{2}\mathrm{dxdydz}}{{∭}_{\mathrm{unit}\;\mathrm{cell}}{\left|E\left(x,y,z\right)\right|}^{2}\mathrm{dxdydz}}$$

where Γ_c_ = Γ_slot_ + Γ_gap_ + Γ_uc._

**Figure 5:**
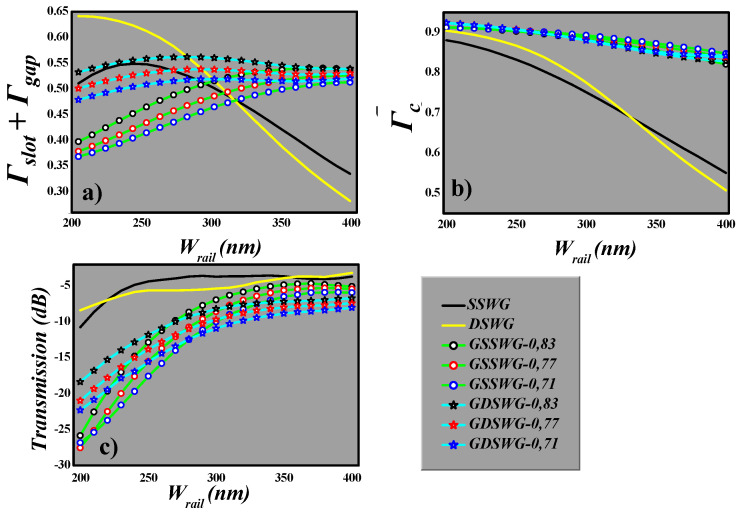
Variation of (**a**) Γ_slot_ + Γ_gap_, (**b**) *Γ_c_*, (**c**) Transmission (dB), on the WG width (*W_rail_*).

In Figure 5a, the mode power confinement in all the four WG schemes is presented. For SSWG and DSWG, the mode confinement is the intensity integration in one slot for SSWG or two slots for DSWG. However, in the case of GSSWG and GDSWG, the mode power in the gaps between silicon grating segments is also considered, as mentioned above. It can be seen that SSWG and DSWG possess maximum mode confinement at W_rail_ = 200 nm, which falls rapidly as W_rail_ increases due to the formation of dielectric mode in the silicon rail. This suggests that the performance of these WGs are strictly dependent on their dimension. Small fabrication error can reduce the mode power, which results in the low overlap integral, as shown in Figure 5b. However, these WGs have relatively low transmission loss (~3 dB to 10 dB) as compared to SWG WGs, which is calculated using the expression: 10 *× log (P_out_/P_in_*), as shown in Figure 5c.

On the other hand, three different duty cycles (0.83, 0.77 and 0.71) are selected for GSSWG and GDSWG to calculate the mode confinement, overlap integral and transmission versus W_rail_ in the range of 200 to 400 nm. It can be seen that mode confinement and overlap integral is least affected by variation in W_rail_. The maximum mode confinement and overlap integral of 0.56 and 0.92 are obtained when W_rail_ is in the range of 200 to 250 nm, respectively. The transmission loss of SWG WGs is relatively high compared to standard slot WGs. However, these losses can be reduced by increasing W_rail_ from 200 to 400 nm. The transmission loss of ~6 dB can be obtained at W_rail_ = 400 nm, however, at the cost of low overlap integral which leads to lower sensitivity.

## 4. Towards Highly Sensitive SWG Racetrack Ring Resonator Design

**Figure 6:**
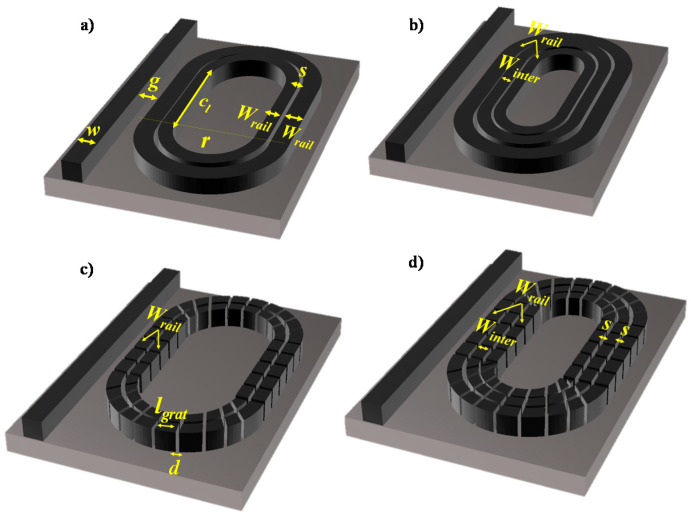
Schematic of race track resonator based on (**a**) SSWG, **(b)** DSWG, (**c**) GSSWG, (**d**) GDSWG.

Refractive index sensors demonstrate several applications in the biological and chemical fields and have been widely studied in recent years, such as solution concentration and pH value, which can be estimated based on refractive index change. The measurement of changes in resonance wavelength is the most common interrogation method in ring resonators. In this section, we studied four configurations of racetrack ring resonators based on different slot WG schemes such as SSWG, DSWG, GSSWG and GDSWG, as shown in Figure 6. In all the four cases, the ring resonator is side coupled to a standard strip WG. In Figure 6a,b, ring resonator designs based on SSWG and DSWG are presented, respectively, whereas in Figure 6c,d, GSSWG and GDSWG race track ring resonators are presented. The period (ᴧ) of the grating WG is fixed at 300 nm with η = 0.83. For a fair analysis, the coupling length (*c_l_*) of all four ring resonator designs is fixed to 3000 nm. For a better understanding of the device design, the geometric parameters are stated in Table 2.

From the E-field distribution profiles presented in Figure 4, it is quite evident that in contrast to the evanescent field on the top surface and sidewalls of the WG, there is a considerably stronger mode field existing on the light propagation path between silicon grating segments. This gives SWG-based microring biosensors an extended surface sensing region on the propagation path, and thus, a distinctive advantage in surface sensing over microrings based on standard WGs. Therefore, surface sensitivity is an important figure of merit. In a resonance-based sensing method, surface sensitivity *S_s_* can be defined as the resonance wavelength shift per the change of surface layer thickness [27].

(4) $$S_{s}=\frac{\Delta \lambda }{\Delta t}=\frac{\lambda }{n_{g}}\left(\frac{\partial n_{eff}}{\partial t}\right)$$

where *n_g_* is group index and *t* is the thickness of the surface layer. The detailed analysis of surface sensitivity can be found in reference [15]. However, in this work, we have studied the bulk sensitivity of the ring resonators based on the WG schemes studied in this paper. 

**Figure 7:**
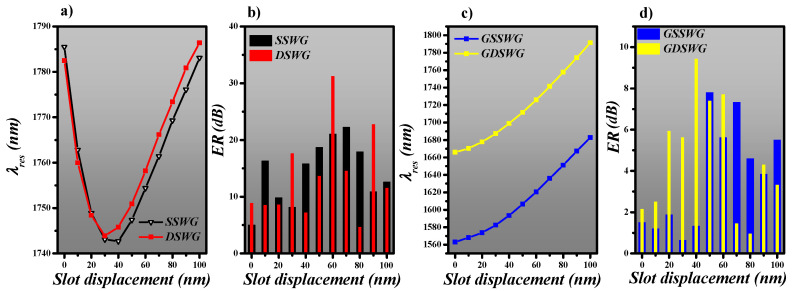
Determination of resonance wavelength of (**a**) SSWG and DSWG, (**b**) GSSWG and GDSWG. Extinction ratio (*ER*) of (**c**) SSWG and DSWG, (**d**) GSSWG and GDSWG.

Before analyzing the sensing capability of the proposed sensor design, the resonance condition and extinction ratio (*ER*) is determined by displacing the slot towards the inner periphery of the ring. ER is calculated using the following expression:

(5) $$ER=10\times log\frac{P_{out}}{P_{in}},$$

where *P_out_* and *P_in_* are the output and input power at *λ_res_,* respectively. The W_rail_, *s* and *g* are maintained at 200, 50 and 100 nm, respectively. The slot is displaced towards the inner periphery of the ring with a step size of 10 nm, which helps to find the optimal position where the maximum mode confinement in the ring is obtained at λ_res_. The intensification in mode power is due to the maximum overlap of the evanescent tail of the propagating mode in the slot. The resonance wavelength and *ER* of SSWG and DSWG based ring resonator designs are plotted in Figure 7a,b, respectively. The *λ_res_* performs a blueshift with slot displacement increases from 0 to ~30 nm. However, slot displacement greater than ~40 nm results in a redshift of *λ_res_*. The *ER* has a significant impact on the placement of the slot, which is evident in Figure 7b. The *ER* of <10 dB is obtained for both symmetric SSWG and DSWG based ring resonator design. However, an optimized slot displacement of 60 nm results in a high *ER* of 22 and 31.2 dB for SSWG and DSWG based ring resonators, respectively.

Figure 7c,d present the λ_res_ and *ER* of GSSWG and GDSWG based ring resonator designs, respectively. The *λ_res_* performs a redshift with an increasing slot displacement towards the inner periphery of the ring, as shown in Figure 7c. As these WGs are refractive index modified and silicon grating segments are periodically arranged along the propagation direction of the light, that is why the mode coupling at *λ_res_* is weaker than a standard WG design. This reduces the *ER* of the ring resonators based on SWG WGs, but is still high enough to interpret the resonance dips in the spectrum. The maximum ER of 7.8 and 9.4 dB is obtained for GSSWG and GDSWG, respectively, as shown in Figure 7d.

## 5. Sensor Performance

The optical resonances are acquired by filling the medium with a material of n = 1.0002–1.0005, which is equivalent to the refractive index of several toxic gases, such as CO_2_, CH_4_ and CO, etc. The most rigorous method is a full 3D FEM approach. Nevertheless, for large structures, this approach is computationally very exhausting and for that reason, not appropriate for large parameter sweeps. Therefore, the transmission spectrum and E-field distribution are simulated using 2D-FEM. Mode sensitivity analysis of SSWG, DSWG, GSSWG and GDSWG presented in the previous section suggests that ring resonators based on SWG WGs can boost the mode power in the upper cladding. Therefore, elevated sensitivity can be expected. High sensitivity is always attractive in these sensors, which strongly depends on light polarization, optical loss and the light–matter interaction. Sensitivity is calculated by using the following expression:

*S* = Δ*λ*/Δ*n*, (6)

where Δ*λ* represents the shift of the sensor resonance in nm and Δ*n* is the difference of the RI in the medium. The microring resonators based on SWG WGs were first demonstrated with bulk sensitivity (Δ*λ_res_/*Δ*n*) of 400–500 nm/RIU [28], which is several times higher than conventional microring resonators based on strip WGs. The figure of merit (*FOM*) is another parameter which should also be considered while designing the ring resonator sensor. *FOM* is expressed as *S/FWHM*, where *FWHM* is full width at half maximum. Chrostowski et al. [29] suggested the intrinsic limit of detection (*iLOD*) as a figure of merit independent on readout circuitry and data processing, which is expressed as $iLOD=\frac{\lambda_{res}}{S\;ΧQ-factor}$ and reflects the detection capabilities of change in refractive index. The *Q-factor* is defined as *λ_res_/FWHM*. Integrated resonators with high *Q-factors* are particularly advantageous for a wide range of applications such as narrow band width filters, high performance lasers, high-efficiency non-linear optic devices and high sensitivity sensors.

**Figure 8:**
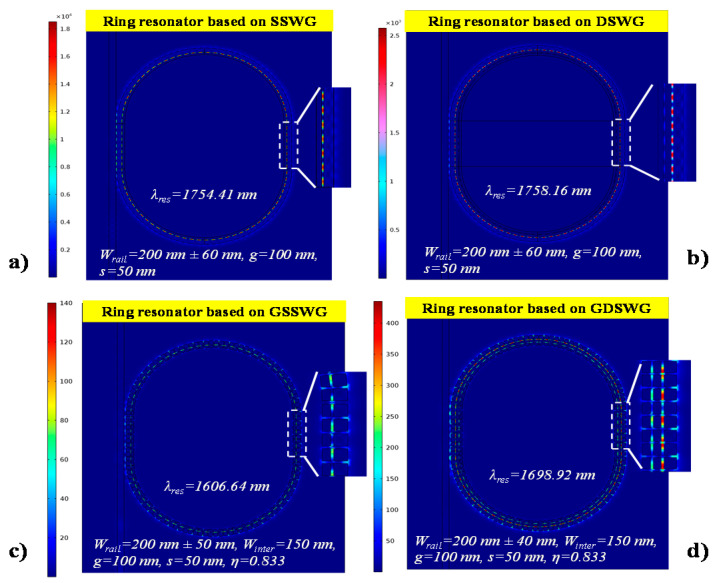
E-field distribution in (**a**) SSWG resonator, (**b**) DSWG resonator, (**c**) GSSWG resonator, (**d**)GDSWG resonator. The inset shows the zoomed section of the ring resonator at *λ_res_*.

The E-field distribution in SSWG, DSWG, GSSWG and GDSWG based racetrack ring resonators is shown in Figure 8a–d. For each design, the optimal slot displacement value is selected, where the maximum *ER* is obtained as labeled in Figure 8. It can be seen in Figure 8c,d, the E-field is prominently enhanced in the grating segment, which provides a strong light–matter interaction. The ring resonators are highly responsive to the ambient refractive index. A slight change in the refractive index can lead to a significant shift in the resonance wavelength (*λ_res_*).

**Figure 9:**
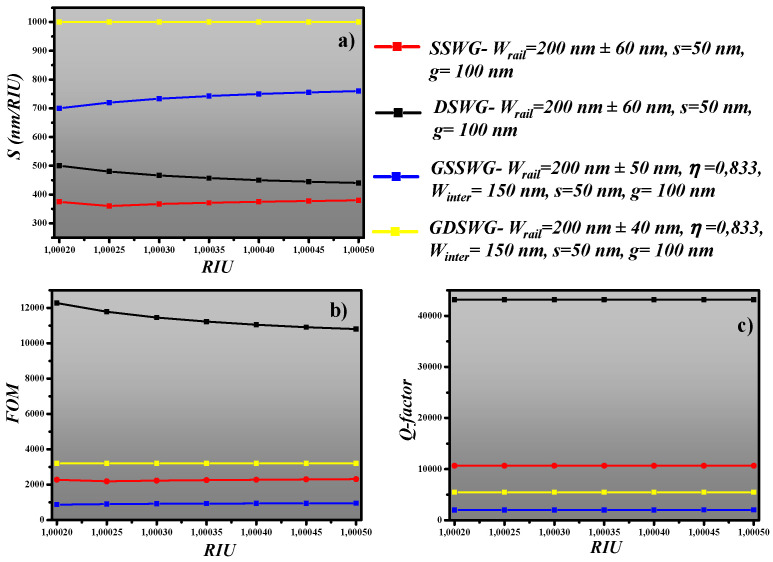
(**a**) Sensitivity, (**b**) *FOM*, (**c**) *Q-factor* of all four designs.

The *S*, *FOM* and Q-factorof all the designs are calculated and displayed in Figure 9. It can be seen from Figure 9a that the GDSWG based resonator has almost 2.5 times higher sensitivity than the resonator design based on the standard SSWG. The narrow *FWHM* of the DSWG ring resonator obtained at *λ_res_* is ~0.04 nm (at optimized WG geometry). Therefore, the *FOM* of the ring resonator design based on DSWG is 12,270, which is higher than the remaining three sensor designs, as shown in Figure 9b). Q-factor helps quantify the losses in the resonator. The sensor designs based on SSWG and DSWG show a high Q-factor of 10,668 and 43,150 at optimized WG parameters, respectively. The *iLOD* of SSWG, DSWG, GSSWG and GDSWG based sensor designs are 4.33 × 10^−4^ RIU, 8.15 × 10^−5^ RIU, 1.05 × 10^−3^ RIU, 3.12 × 10^−4^ RIU, respectively.

In Table 3, we have listed several recently proposed and demonstrated refractive index sensors based on an SOI platform. However, our main proposed designs based on GSSWG and GDSWG outclass all the previous reports [5,24,28,30,31,32,33,34,35,36,37] with an exceptionally high sensitivity of 760 and 1000 nm/RIU (at optimized parameters), respectively. The parameters used in our study are practical and achievable with existing standard CMOS fabrication technology.

## 6. Concluding Remarks

In this paper, a novel design of a racetrack ring resonator composed of the subwavelength grating double slot waveguide is proposed for gas sensing application. Different configurations of slot waveguides, such as single slot waveguide, double slot waveguide, subwavelength grating single and double slot waveguides, are studied and compared via the finite element method. Subwavelength grating slot waveguides are a special type of optical waveguides, where quasi-TE mode experiences high disruption at the interface between the low index region (slot) and gaps between the silicon segment result in high electric field intensity. This characteristic makes subwavelength grating waveguides a promising candidate for applications that involve strong light–matter interaction, such as sensing and non-linear photonics. The sensing capability of the device can be significantly enhanced, which is not possible to attain with conventional slot waveguides. Our proposed design is capable of providing a sensitivity of 1000 nm/RIU, which is approximately 2.5 × higher than the values that can be obtained via standard slot waveguide ring resonators of the same geometric parameters. The transmission loss of subwavelength grating waveguides can be significantly high if not designed properly. Therefore, there is always a compromise between sensitivity and *Q-factor*. The waveguide dimensions at which the sensor device is highly sensitive can offer a low *Q-factor* ~5445. The *FOM* and *Q-factor* of such devices can be improved by increasing the width of the waveguide at the cost of reduced sensitivity. On the other hand, the ring resonator based on DSWG offers a maximum *Q-factor* and limit of detection of 43,150 and 8.15 × 10^−5^ RIU, respectively.

## Figures and Tables

**Table 1:** Geometric parameters of WGs.

For All Four WG Schemes	SSWG and GSSWG	For All Four WG Schemes	DSWG and GDSWG	GSSWG and GDSWG	GSSWG and GDSWG	DSWG and GDSWG
*H_rail_* (nm)	*W_rail_* (nm)	*s* (nm)	*W_rail_* (nm)	*l_grat_* (nm)	*d* (nm)	*W_inter_* (nm)
220	200–400	50	200–400	250	50–100	150

**Table 2:** Race track resonator parameters.

WG Type	*w* (nm)	*g* (nm)	*W_rail_* (nm)	*s* (nm)	*Slot Displacement* (nm)	*W_inter_* (nm)	*c_l_* (nm)	*l_grat_* (nm)	*d* (nm)	*r* (nm)
SSWG	400	100	200	50	0–100	-	3000	-	-	5000
DSWG	400	100	200	50	0–100	150	3000	-	-	5000
GSSWG	400	100	200	50	0–100	-	3000	250	50	5000
GDSWG	400	100	200	50	0–100	150	3000	250	50	5000

**Table 3:** Previously reported sensitivities of SOI ring resonators.

No.	Resonator Designs	Sensitivity (nm/RIU)	Reference
1	Strip WG ring resonator	100	24
2	SWG strip WG ring resonator	400–500	28
3	Bragg grating slot WG	340	30
4	Strip WG ring resonator	439	31
5	Slot WG ring resonator	212.1	32
6	Single semiconductor nanowire	235	33
7	Wire WG ring resonator	135	34
8	Strip WG ring resonator	270	35
9	Silicon microring	222	36
10	SWG double slot microring	840	37
11	Slot WG ring resonator	298	5
12	SSWG racetrack ring resonator	380	This work
13	DSWG racetrack ring resonator	500	This work
14	GSSWG racetrack ring resonator	700–760	This work
15	GDSWG racetrack ring resonator	1000	This work
